# Specific GAG ratios in the diagnosis of mucopolysaccharidoses

**DOI:** 10.1002/jmd2.12412

**Published:** 2024-02-08

**Authors:** Déborah Mathis, Jean‐Christophe Prost, Gabriela Maeder, Liya Arackal, Haoyue Zhang, Sandra Kurth, Katrin Freiburghaus, Jean‐Marc Nuoffer

**Affiliations:** ^1^ University Institute of Clinical Chemistry, Inselspital, Bern University Hospital, University of Bern Bern Switzerland; ^2^ Biochemical Genetics Laboratory Duke University Health System Durham North Carolina USA; ^3^ Department of Pediatrics, Division of Pediatric Endocrinology and Inborn Errors of Metabolism University Children's Hospital Bern Bern Switzerland

**Keywords:** chondroitin sulfate, dermatan sulfate, diagnosis, dimethylmethylene blue dye‐binding (DMB) assay, GAG, glycosaminoglycans, heparan sulfate, keratan sulfate, LC–MS, MPS, mucopolysaccharidosis, ratios, reference values, urinary tract infection

## Abstract

Mucopolysaccharidoses (MPS) screening is tedious and still performed by analysis of total glycosaminoglycans (GAG) using 1,9‐dimethylmethylene blue (DMB) photometric assay, although false positive and negative tests have been reported. Analysis of differentiated GAGs have been pursued classically by gel electrophoresis or more recently by quantitative LC–MS assays. Secondary elevations of GAGs have been reported in urinary tract infections (UTI). In this manuscript, we describe the diagnostic accuracy of urinary GAG measurements by LC–MS for MPS typing in 68 untreated MPS and mucolipidosis (ML) patients, 183 controls and 153 UTI samples. We report age‐dependent reference values and cut‐offs for chondroitin sulfate (CS), dermatan sulfate (DS), heparan sulfate (HS) and keratan sulfate (KS) and specific GAG ratios. The use of HS/DS ratio in combination to GAG concentrations normalized to creatinine improves the diagnostic accuracy in MPS type I, II, VI and VII. In total 15 samples classified to the wrong MPS type could be correctly assigned using HS/DS ratio. Increased KS/HS ratio in addition to increased KS improves discrimination of MPS type IV by excluding false positives. Some samples of UTI patients showed elevation of specific GAGs, mainly CS, KS and KS/HS ratio and could be misclassified as MPS type IV. Finally, DMB photometric assay performed in MPS and ML samples reveal four false negative tests (sensitivity of 94%). In conclusion, specific GAG ratios in complement to quantitative GAG values obtained by LC–MS enhance discrimination of MPS types. Exclusion of patients with UTI improve diagnostic accuracy in MPS IV but not in other types.

**Synopsis**

Specific GAG ratios in complement to individual quantitative GAG values obtained by LC–MS enhance discrimination of MPS types.

## 1 INTRODUCTION

Mucopolysaccharidoses (MPS) are a group of genetic diseases characterized by a deficiency of lysosomal enzymes required for the degradation of glycosaminoglycans (GAG).^1^ MPS includes different types and subtypes, depending on the deficient enzyme and accumulation of GAGs (Table S1).^2^ GAGs are linear highly sulfated polysaccharides constructed from five different disaccharide building blocks, heparan sulfate (HS), dermatan sulfate (DS), chondroitin sulfate (CS), keratan sulfate (KS) and hyaluronic acid (HA), the latter lacking sulfation.^3^ GAGs are contained in proteoglycans that play central roles in extracellular matrix, on cell surface and in cell signaling.^4^

Lysosomal accumulation of GAGs is not only observed in MPS but also in multiple sulfatase deficiency (MSD) as well as mucolipidoses (ML). ML is caused by deficient post‐translational modification of lysosomal enzymes in the Golgi that is essential for enzyme transport into the lysosome.^5^, ^6^ Accumulation of GAGs in tissues and extracellular matrixes observed in MPS and ML leads to typical clinical features as organomegaly, dysostosis multiplex, and coarse facies.^1^ Enzyme replacement therapy (ERT) and hematopoietic stem cell transplantation (HSCT) are conventional treatments for some MPS types.^7^ They had proven efficacy, especially if administered early in the disease course before affection of major organs.^8^ Thus, early diagnosis is essential to hamper or delay progression of the disease.^9^

Screening for MPS is usually performed by biochemical assessment of urinary GAG excretion.^10^ This can be achieved by measurement of total GAGs with typical colorimetric methods as the 1,9‐dimethylmethylene blue dye‐binding (DMB) photometric assay^11^ in combination with measurement of differentiated GAGs by gel electrophoresis (ELP), thin‐layer chromatography (TLC) or liquid chromatography–tandem mass spectrometry (LC–MS).^3^, ^12^, ^13^, ^14^, ^15^, ^16^, ^17^ Differentiation of GAGs by LC–MS implies either the determination of the disaccharide building blocks DS, CS, HS, and KS after hydrolysis (chemically or enzymatically) or are based on the detection of non‐reducing ends of endogenous oligosaccharides present in urine.^18^, ^19^, ^20^, ^21^ Performing total and differentiated assays simultaneously is still of use in most laboratories,^10^ although it has been suggested that LC–MS measurement should replace other methodologies (DMB, TLC, ELP).^14^

In this manuscript, we describe the diagnostic accuracy of urinary GAG measurements (CS, DS, HS, and KS) by LC–MS for MPS typing in a cohort of 68 untreated MPS and ML patients, 183 controls and 153 samples from patients with urinary tract infection (UTI). (1) We determine age‐dependent reference values. (2) We determine if diagnostic accuracy improves by using specific ratios in combination to GAG concentrations normalized to creatinine (GAG/Cr). (3) We studied the consequences of UTI on GAG excretion. (4) We compared the LC–MS results to total GAGs obtained by DMB photometric assay. (5) Lastly, we discuss possible pitfalls of the method.

## 2 METHODS

### 2.1 Patients and samples

All samples used in this study were fully anonymized and originated from left over material from our institute or Metabolic University centers in Lausanne, Geneva, Zürich, Vienna and were given to us for research purposes without any further conditions. External quality insurance samples from ERNDIM urine MPS schemes (2016–2022) were also used in this study with permission of ERNDIM. In total 68 urine samples from individual untreated patients with MPS and ML were available. Sample data included: age at sample collection, gender, MPS type and therapy. All samples were shipped under dry ice and stored at −80°C until analysis. Anonymized control urines (*n* = 183) and urines from urinary tract infection (UTI, *n* = 153) were left‐over samples from routine diagnostics of the center of laboratory medicine of our Hospital. Urine dipstick analysis were determined for all left‐over samples and sorted as controls if negative for leucocytes, nitrites and bacteria, or sorted as UTI if one of those fields was positive. GAG concentrations in urine were normalized to urinary creatinine (GAG/Cr), which was determined using an enzymatic method (Cobas c 702, Roche diagnostics).

### 2.2 LC–MS measurements

The LC–MS method for the determination of CS, DS, HS and KS is based on the protocols of Zhang et al.^12^ and Auray‐Blais et al.^15^ with small adaptations as described in the supplementary Information. Analysis was performed on an Acquity I‐Class system coupled to triple quadrupole MS (Xevo TQ‐S Waters, Milford, MA, USA) mounted with an Acquity UPLC BEH amide column (2.1 × 50 mm^2^, 1.7 μm). CS, DS and HS were ionized by electrospray ionization (ESI) in positive mode, whereas negative mode was used for KS. Further parameters are given in the supplementary Information (Table S2). Data were acquired with MassLynx (version 4.1 Waters) and processed with TargetLynx.

### 2.3 DMB photometric assay

Total GAG concentrations were measured using a modified protocol based on previous work^11^ as described in the supplementary Information. Results are expressed in g/moL creatinine and evaluated in age dependency according to Table S3.

### 2.4 Data analysis

Data analysis and statistics were performed in GraphPad Prism 9.1.0. The Mann–Whitney‐*U* test for non‐parametric unpaired data was used to analyze differences between groups. A *p*‐value of <0.05 was considered statistically significant. Ratio to cut‐off value was calculated as GAG/Cr concentration divided by the age‐dependent cut‐off value. A “ratio to cut‐off value” higher than 1 indicates increased GAG/Cr value.

## 3 RESULTS

### 3.1 Age‐dependent GAG reference values of DS, CS, HS, and KS by LC–MS

**FIGURE 1:**
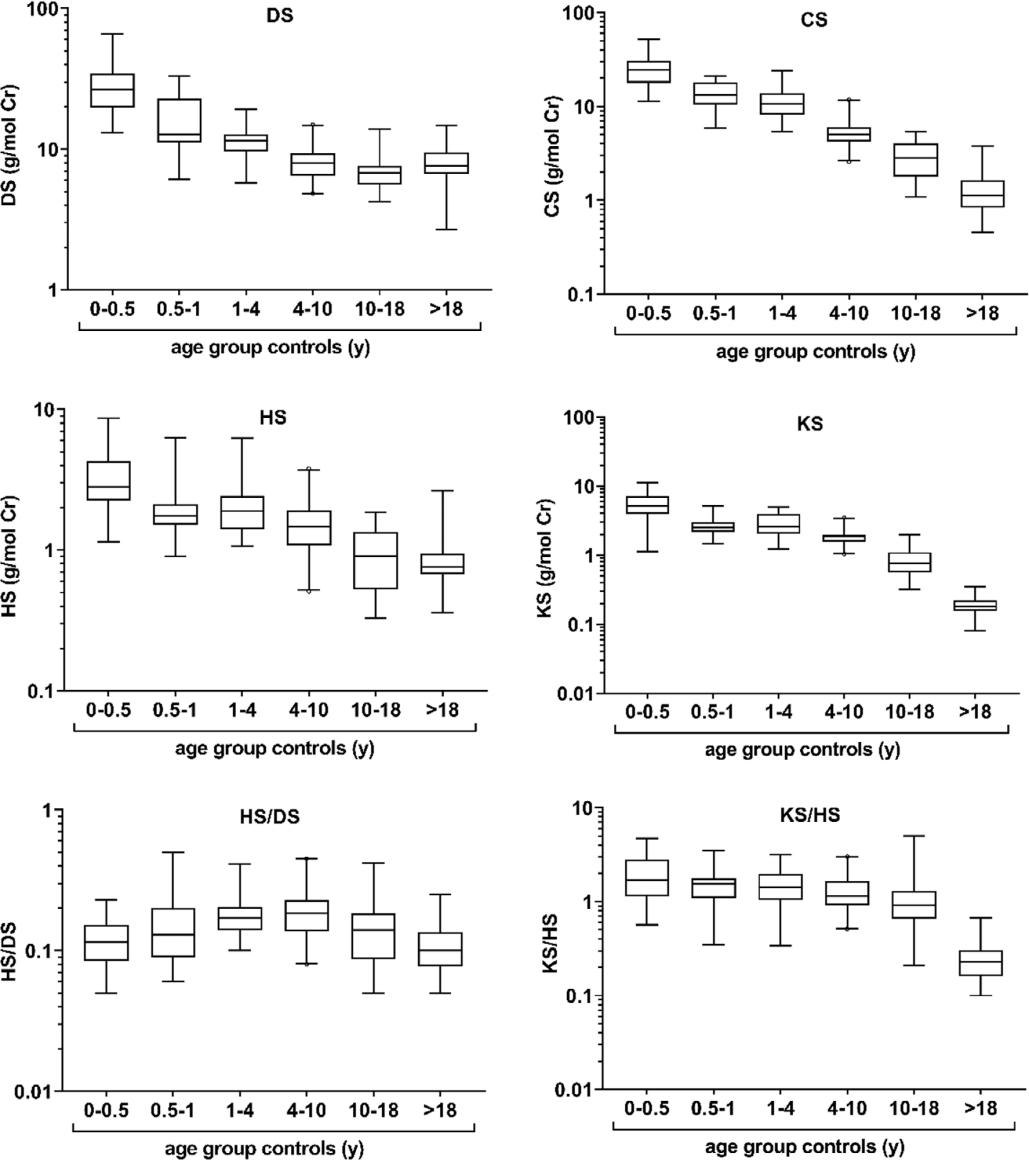
Derived age‐dependent reference values of dermatan sulfate (DS), chondroitin sulfate (CS), heparan sulfate (HS), and keratan sulfate (HS) as well as two specific ratios HS/DS and KS/HS. Boxplots depict the median and quartiles and whiskers the 2.5–97.5 percentiles. The sample sizes for different age groups are: *n* = 31 (0–0.5 y); *n* = 20 (0.5–1 y); *n* = 26 (1–4 y); *n* = 42 (4–10 y); *n* = 34 (10–18 y); *n* = 30 (>18 y).

Age‐dependent reference values for DS, CS, HS, KS and two specific ratios HS/DS and KS/HS has been derived by measuring a total of 183 left‐over urine samples from different age by LC–MS (Figure 1). Median and 97.5 percentile for each age group is given in Table S4. All individual GAG/Cr decrease with age, as previously described.^12^, ^15^, ^17^, ^22^ The cut‐off value for HS/DS ratio is independent of age and was chosen at 0.5 for all groups, which was the highest value detected in controls. The age groups were chosen in accordance to published data from Zhang et al.^12^

### 3.2 Specific GAG ratios in combination to GAG/Cr concentrations improve diagnostic accuracy in MPS and ML patients

**FIGURE 2:**
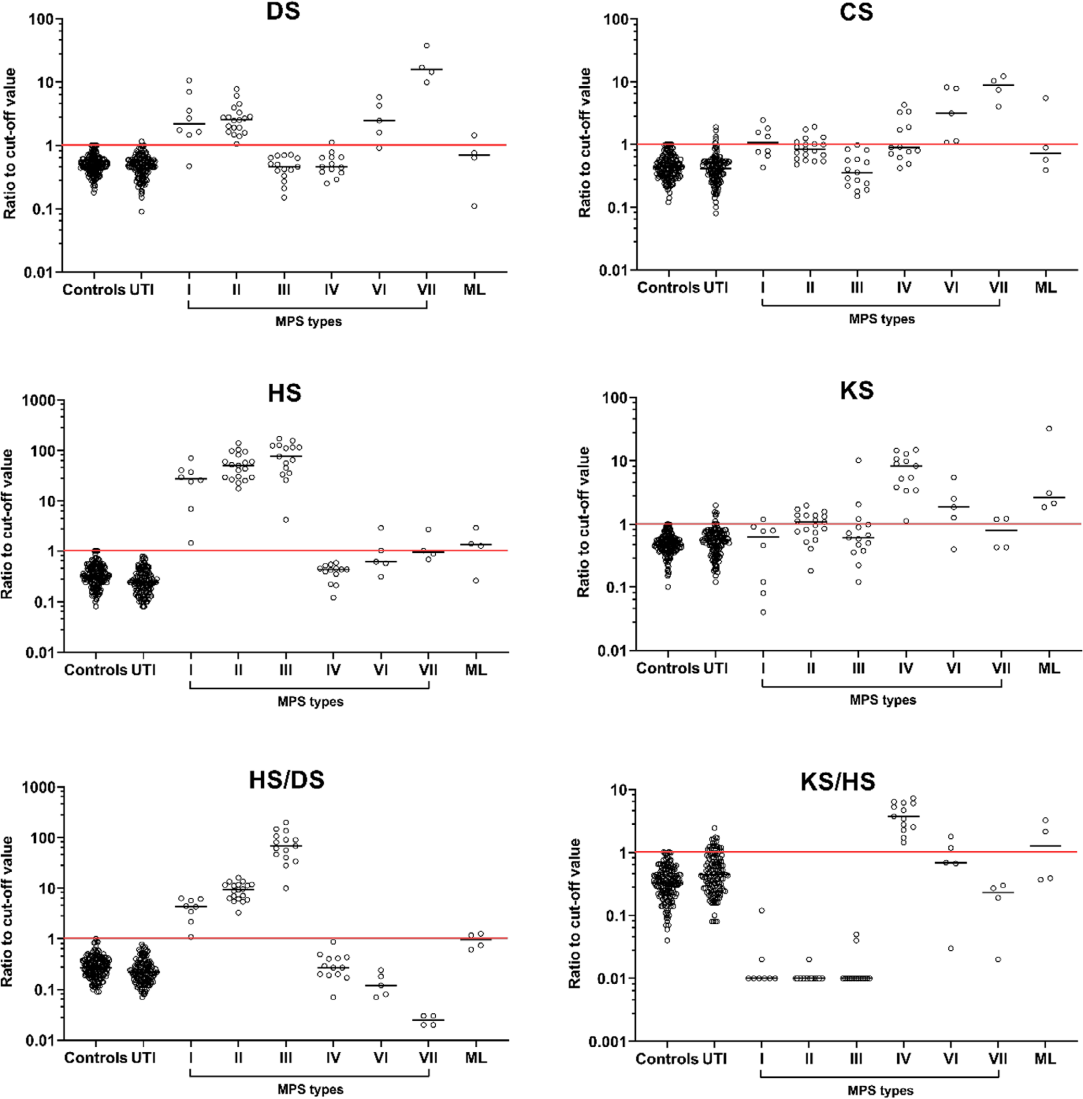
Ratio to cut‐off values (>97.5 p) of dermatan sulfate (DS), chondroitin sulfate (CS), heparan sulfate (HS), and keratan sulfate (HS), as well as specific ratios HS/DS and KS/HS in urine samples of controls (*n* = 183), urinary tract infection (UTI, *n* = 153), and untreated patients (*n* = 68) with mucopolysaccaridosis (MPS) type I, II, III, IV, VI, VII and mucolipidosis (ML). UTI: urinary tract infection.

Differential GAG pattern obtained by LC–MS of controls (*n* = 183), UTI (*n* = 153), and MPS and ML patients (*n* = 68) are displayed in Figure 2. HS/DS and KS/HS were the most discriminative ratios for MPS typing and thus discussed hereafter. DS/CS and KS/DS were not discriminative for any MPS type. HS/CS ratio displays a similar pattern than HS/DS but was not discriminative for MPS I & II against MPS III (Figure S1).

MPS type I and II are characterized by elevation of DS, HS, and HS/DS ratio. Both types display the same GAG pattern and are thus treated as a group hereafter. MPS type III is characterized by elevation of HS with normal DS and a significant elevation of HS/DS ratio (*p* < 0.0001). MPS type IV present elevation of KS and KS/HS ratio. MPS type VI and VII exhibit increased DS and CS concentrations with normal or low HS/DS ratio and cannot be differentiated in this work probably due to limited number of data. All ML samples (*n* = 4) showed elevation of KS, three samples elevation of HS and one sample elevation of all four individual GAGs (Figure 2).

Diagnostic accuracy for MPS typing using GAG/Cr concentrations only versus specific GAG ratios in combination to GAG/Cr were calculated (Table 1). The use of increased HS/DS ratio in addition to increased DS and HS lead to better discrimination of MPS I and II versus other types. Three false positives could be eliminated adding increased HS/DS ratio (one MPS VI, one MPS VII and one ML, Table 1). Furthermore, the level of HS/DS serve as further tool to differentiate MPS III from MPS I and II. Normal HS/DS ratio serve in the differentiation of MPS VI and VII. Number of false positives decreased from 13 to 1 adding normal HS/DS ratio as further criteria (Table 1). Moreover, HS/DS ratio lower than 0.02 seems to be specific for MPS type VII but this finding needs to be validated with increased sample size (Figure 2).

**TABLE 1:** Diagnostic accuracy using GAG/Cr concentrations only and GAG/Cr in combination to specific ratios for mucopolysaccharidoses (MPS) typing.

	MPS I and II		MPS III		MPS IV		MPS VI and VII		Classed as MPS or ML
	DS↑ HS↑	DS↑ HS↑ HS/DS↑	DS n HS↑	DS n HS↑ HS/DS↑	KS↑	KS↑ KS/HS↑	DS↑ CS↑	DS↑ CS↑ HS/DS n	
True positive	26	26	15	15	13	13	8	8	68
True negative	374	377	386	386	359	384	382	394	323
False positive	3^a^	0	3^b^, ^c^	3^b^, ^c^	32^d^	7^e^	13^f^, ^g^	1^f^	13^i^
False negative	1^b^	1^b^	0	0	0	0	1^h^	1^h^	0
Sensitivity (%)	96	96	100	100	100	100	89	89	100
Specificity (%)	99	100	99	99	92	98	97	100	96
PPV (%)	90	100	83	83	29	65	38	89	84
NPV (%)	100	100	100	100	100	100	100	100	100
Diagnostic accuracy (%)	99	100	99	99	92	98	97	100	97

*Note*: The data includes controls (*n* = 183), UTI samples (*n* = 153) and samples of untreated MPS or ML patients (*n* = 68) with a total of 404 samples.

^a^ Criteria also fulfilled by one MPS VI, one MPS VII, and one ML.

^b^ One MPS I with normal DS wrongly classed as MPS III.

^c^ Criteria also fulfilled by two ML.

^d^ Criteria also fulfilled by 21 samples MPS all types, 2 ML, and 9 UTI.

^e^ Criteria also fulfilled by two MPS VI, two ML, three UTI.

^f^ Criteria also fulfilled by one MPS IV.

^g^ Criteria also fulfilled by 11 MPS I and II and 1 ML.

^h^ One MPS type VI with DS normal and not assignable to any type, but not normal.

^i^ Criteria also fulfilled by 13 UTI samples.

Increased KS/HS ratio in addition to increased KS improves discrimination for MPS type IV. Number of false positives was reduced from 32 to 7 (false positives: 21 samples of any MPS types, 2 ML, and 9 UTI, Table 1). Exclusive elevation of KS/HS ratio was unspecific and classified as normal.

In our dataset regardless of using ratios or GAG/Cr only, we found two MPS samples, which were either assignable to the wrong type or not clearly assignable. One MPS type I urine sample had normal DS concentration and thus fulfilled wrongly the criteria of MPS type III. One MPS type VI sample had normal DS but elevation of CS and KS and could not be assigned to a specific MPS type. ML samples were hardly assignable. Importantly, all 68 pathological samples were classified as MPS or ML, as no samples displayed normal values.

### 3.3 Urinary tract infection (UTI) urine samples

153 left‐over urine samples tested positive for leucocytes, nitrite or bacteria by urine‐status (dipsticks) were analyzed with the LC–MS method. Elevations of DS, CS, and KS were detected in 1, 6 and 9 samples, respectively, with CS and KS concomitantly raised in 3 samples leading to 13 samples with increased GAG/Cr concentrations. Elevation of KS/HS ratio was detected in 21 UTI samples. Three of those samples exhibited increased KS as well, thus fulfilling MPS IV criteria (Table 1). The sole increase of KS/HS ratio without elevation of KS was classed as normal, as no MPS samples exhibited this pattern. The 10 remaining samples with slight increased GAG/Cr were false positives for MPS, however they presented with unspecific patterns not assignable to any explicit MPS type.

### 3.4 Urinary total MPS from DMB photometric assay

Urine samples from confirmed MPS and ML patients (*n* = 68) were analyzed using the DMB photometric assay. Total MPS were found normal in one MPS III and three MPS IV affording a sensitivity of 94% (Figure S2). Controls and UTI samples of this study were not analyzed with the DMB photometric assay.

## 4 DISCUSSION

Diagnosis of MPS and MPS typing remains challenging although years of experience have passed.^23^ We describe in here GAG patterns of CS, DS, HS and KS in urine samples of one of the largest cohort of MPS and ML patients analyzed by LC–MS so far. We show that the use of specific ratios in combination to GAG/Cr concentrations lead to better differentiation of the MPS types.

Increased DS is observed in MPS type I, II, VI, and VII with increased HS observed in MPS type I, II, and III.^10^, ^15^ We show that the use of HS/DS ratio unambiguously helps in the distinction of those different types, although not to differentiate type I from II. Langerei et al.^16^ plotted the DS/HS ratio of MPS I and II and show differences between the two types. The same trend was observed in our data (opposite as we plotted HS/DS) but no clear differentiation could be determined.

KS/HS ratio in combination to increased KS is specific for MPS IV, though those criteria were also found in MPS VI, ML and in some patients with UTI. Thus, false positives resembling MPS type IV are detected by LC–MS, while to a lesser extend considering KS/HS ratio compared to KS elevation only. Exclusion of patients with UTI improved diagnostic accuracy of MPS type IV, however not MPS of other types.

The age‐dependent reference values obtained by LC–MS are comparable with the published values by Zhang et al.^12^ for CS and HS, with the results from Auray‐Blais et al.^15^ for DS and Auray‐Blais et al.^22^ for KS (Table S5). The sample preparation technique, that is, methanolysis or enzymatic reaction has an important impact on reference values reported. Verheyen et al.^16^ used enzymatic digestion for the hydrolysis of HS, DS and KS and report different values.

The DMB photometric assay is frequently used as screening method in patients with suspected lysosomal storage disorders, however it should not be used on its own.^10^ It is fast, cheap, uncomplicated and easily available in contrast to the LC–MS analysis or gel electrophoresis. In our cohort of 68 untreated MPS and ML patients, we identified three MPS IV and one MPS III patient samples that had normal total GAGs (sensitivity 94). In the LC–MS assay, those samples exhibited clear MPS patterns. Such normal findings by DMB photometric assay in MPS patients type III and IV have been previously reported.^10^, ^16^, ^24^ Hence, any negative result obtained by DMB assay should be considered with caution and further investigations continued especially if clinical suspicion of MPS persists.^10^, ^25^, ^26^

Diagnosis of MPS and ML remains tedious and despite the fact that the diagnostic accuracy of the LC–MS/MS method approached 100% using ratios, it is still complex, expensive and subjected to pitfalls and interferences. Thus, alternative LC–MS technologies based on the determination of endogenous, non‐reducing end oligosaccharides^18^, ^19^, ^20^, ^21^ are also used and may facilitate diagnosis of MPS.

Heparin interfers with the quantification of heparan in the LC–MS assay because the two polysaccharides share the same repeating domains. A patient sample with suspicion of MPS revealed increased total GAGs by DMB assay, as well as a significant elevation of HS in the LC–MS analysis (data not shown). In this patient however, the gel electrophoresis was normal. On further investigations, the patient showed normal activity of lysosomal enzymes and GAG elevations were related to heparin therapy.

We analyzed urine samples of two MPS type X patients described by Verheyen et al.^27^ and found normal total GAGs by the DMB photometric assay, as well as normal pattern by LC–MS (data not shown). They previously have reported elevation of DS in two urine samples analyzed by multiplex LC–MS assay^16^ and normal pattern by DMB photometric assay and electrophoresis.

In conclusion, specific GAG ratios in complement to individual quantitative GAG/Cr values obtained by LC–MS allow a better discrimination between the different MPS types. HS/DS ratio is elevated in MPS I, II, and even more strikingly in MPS type III but normal or decreased in MPS VI and VII, respectively. KS/HS is useful to discriminate MPS type IV in combination with increased KS. Exclusion of patients with UTI improves diagnostic accuracy of MPS type IV, however not diagnostic accuracy of other MPS types. We still recommend excluding urine samples with signs of infection for GAG analysis by LC–MS.

## AUTHOR CONTRIBUTIONS

**Déborah Mathis:** Compiled all data and results, drafted figures and wrote the manuscript. **Jean‐Christophe Prost:** Supervised the set‐up of the LC–MS method, analyzed all data and drafted. **Gabriela Maeder:** Provided technical support and analyzed samples. **Liya Arackal:** Set up the LC–MS method and analyzed samples with LC–MS and DMB assay. **Haoyue Zhang:** Provided technical support in establishing the LC–MS method. **Sandra Kurth:** Provided support for the DMB assay. **Katrin Freiburghaus:** Search the literature and initiated the set‐up of the LC–MS method. **Jean‐Marc Nuoffer:** Collect samples, initiated and supervised the study. All authors revised and approved the final version of the manuscript.

## FUNDING INFORMATION

This study was supported by the Batzebaer Stiftung Font of the University Children's Hospital Bern, Switzerland.

## CONFLICT OF INTEREST STATEMENT

Déborah Mathis, Jean‐Christophe Prost, Gabriela Mäder, Liya Arackal, Haoyue Zhang, Sandra Kurth, Katrin Freiburghaus, and Jean‐Marc Nuoffer declare that they have no conflict of interest.

## PATIENT CONSENT STATEMENT

Samples were fully anonymized. All procedures followed were in accordance with the ethical standards of the responsible committee on human experimentation (institutional and national) and with the Helsinki Declaration of 1975, as revised in 2000 (5).

## ETHICS STATEMENT

Ethical approval was not required as the restrospective study was undertaken on fully anonymised leftover urine samples. All procedures followed were in accordance with the ethical standards of the responsible committee on human experimentation (institutional and national) and with the Helsinki Declaration of 1975, as revised in 2000 (5).

## Supporting information


**Data S1.** Supporting Information.

## Data Availability

All data are provided in the manuscript and supplemental information.
