# Supplementary material for: Specific GAG ratios in the diagnosis of mucopolysaccharidoses
Source: JIMD Rep. 2024 Feb 8;65(2):116–23. doi: 10.1002/jmd2.12412 (PMC10910216; doi:10.1002/jmd2.12412)
Supplement: Supplementary file 1 — Data S1. Supporting Information. [file JMD2-65-116-s001.docx]

**Supplementary Material**

**Specific GAG ratios in the diagnosis of mucopolysaccharidoses**

Déborah Mathis^1*^, Jean-Christophe Prost^1^, Gabriela Mäder^1^, Liya Arackal^1^, Haoyue Zhang^2^, Sandra Kurth^1^, Katrin Freiburghaus^1^, Jean-Marc Nuoffer^1, 3^

^1^University Institute of Clinical Chemistry, Inselspital, Bern University Hospital, University of Bern, Bern, Switzerland

^2^Biochemical Genetics Laboratory, Duke University Health System, Durham, NC, USA

^3^Department of Pediatrics, Division of Pediatric Endocrinology and Inborn Errors of Metabolism, University Children's Hospital Bern, Switzerland

**Supplementary Introduction**

**Table S1.** Classification of mucopolysaccharidoses (MPS). Enzyme involved, primary GAG stored, major clinical manifestation and therapeutic options. HS: heparan sulfate; DS: dermatan sulfate; CS: chondroitin sulfate; KS: keratan sulfate; HA: hyaluronic acid; ERT: enzyme replacement therapy; HSCT: hematopoietic stem cell transplantation.

| **MPS Type**  Eponym | **Enzyme**  **defect** | **Primary GAG stored** | **Selected clinical manifestations** | **Therapeutic options** |
| --- | --- | --- | --- | --- |
| **MPS I**  Hurler | α-L-Iduronidase | DS, HS | Corneal clouding, skeletal deformities, coarse facial features, enlarged tongue and head, hepatosplenomegaly, cardiomyopathy, hearing loss, mental retardation | ERT, HSCT |
| **MPS I**  Scheie | α-L-Iduronidase | DS, HS | Corneal clouding, joint stiffness  normal intelligence and stature | ERT, HSCT |
| **MPS II**  Hunter | Iduronate-2-sulfatase | DS, HS | Skeletal deformities, hepatosplenomegaly, cardiomyopathy, respiratory infections, mental retardation | ERT, HSCT |
| **MPS IIIA**  Sanfilippo A | Heparan-N-sulfatase | HS | Behavioural problems, delayed development, severe neurological degeneration, loss of intellectual and motoric skills, mild coarse facial features | - |
| **MPS IIIB**  Sanfilippo B | α-N-Acetyl-glucosaminidase | HS |  |  |
| **MPS IIIC**  Sanfilippo C | α-Glucosaminide-N-acetyltransferase | HS |  |  |
| **MPS IIID**  Sanfilippo D | N-Acetylglucosamine-6-sulfatase | HS |  |  |
| **MPS IVA**  Morquio A | N-acetyl-galactosamine-6- sulfatase | KS, CS | Skeletal dysplasia, joint laxity, corneal clouding, short-trunk dwarfism, neurological abnormalities | ERT, HSCT |
| **MPS IVB**  Morquio B | β-Galactosidase | KS |  | - |
| **MPS VI**  Maroteaux-Lamy | N-Acetyl-galactosamine-4- sulfatase | CS, DS | Dysostosis multiplex, corneal clouding, claw-hand deformities, hepatosplenomegaly, airway infection, normal intelligence | ERT, HSCT |
| **MPS VII**  Sly | β-D-Glucuronidase | CS, HS, DS | Skeletal deformities, coarse facial features, scaphocephaly, hepatosplenomegaly, | ERT, HSCT |
| **MPS IX**  Natowicz | Hyalurono-glucosaminidase 1 | HA | Short stature, periarticular soft tissue, cleft palate, flat nose | - |

**Supplementary Method**

**Chemicals and reagents**

Ultra-pure water was produced in-house using a Milli-Q station from Millipore (Zug, Switzerland). Acetonitrile (ACN), methanol (MeOH), isopropanol LC-MS grade and ammonium acetate ULC/MS–CC/SFC were purchased from Biosolve (Dieuze, France). Acetyl chloride, methanol-d4, 3 mol/L hydrogen chloride in methanol, dermatan sulfate (chondroitin sulfate B sodium salt from porcine intestinal mucosa) and heparan sulfate were obtained from Sigma-Aldrich (Buchs, Switzerland). Chondroitin 6-sulfate sodium salt from shark cartilage was from Fluka (Buchs, Switzerland). Normal human urine was from Golden West Biologicals (Temecula, CA, USA). Sodium keratan sulfate from shark cartilage was obtained from AMS Biotechnology (Abingdon, U.K.). N-acetyl lactosamine 6-sulfate sodium salt (LacNAc (S1)), N-acetyl lactosamine 6,6’-disulfate disodium salt (LacNAc (S2)), N-acetyl lactosamine 6-sulfate sodium salt-^13^C_6_ (LacNAc (S1)-^13^C_6_) and N-acetyl lactosamine 6,6’-disulfate disodium salt-^13^C_6_ (LacNAc (S2)-^13^C_6_) were purchased from GlycoSyn (Lower Hutt, New Zealand).

**LC-MS/MS measurement**

The LC-MS/MS method is based on the protocols of Zhang et al.^1^ and Auray-Blais et al.^2^

In short, DS, CS, HS were chemical cleaved into dimethylated dimers by methanolysis and mixed with internal standard. Deuterated internal standards of CS, DS and HS were synthesized in our lab as described previously. Sample preparation were pursued as follow: 25 µL of calibrator, positive controls or patient urine were pipetted in a vial and dried at 35 °C under nitrogen. 200 µL of 3 mol/L methanolic HCl were added to the dried sample followed by 70 min incubation at 65 °C, then immediately dried again at room temperature under nitrogen. All samples were resolubilised in 200 µL of solvent B and 25 µL IS mix and transferred to HPLC vial for analysis. GAGs were quantified using external calibration curves prepared in urine and injected separately.

Enzymatic digestion using keratanase was performed for the determination of KS, which was analyzed separately from the other GAGs. In short 100 μL of urine, 100 μL IS, 100 μL 0.1 mol/L NH_4_Ac pH7 and 10 μL keratanase (0.02 U/mL) were pipetted with 1.5 mL reaction tubes and incubated at 40 °C for 3 h. After centrifugation at 4°C, 5 min, max force, 100 μL were transferred to HPLC vial for analysis.

Chromatographic analysis was performed on an Acquity I-Class system (Waters, Milford, MA, USA). An Acquity UPLC BEH amide column (2.1 mm x 50 mm, 1.7 µm, Waters, Milford, MA, USA) was maintained at 30 °C. H_2_O:ACN (9:1 v/v) (A) and H2O:ACN (1:9 v/v) (B) buffered with 10 mmol/L ammonium acetate were used as mobile phases at a flow rate of 0.4 mL/min. Injection volume was 1 μL. The elution gradient was: 0.0–0.5 min, 100% B; 0.5–4.5 min, 100–75% B; 4.5–5.5 min, 75–88% B; 5.5–6.0 min, 88–100% B; 6.0–7.0 min, 100% B.

CS, DS, HS and KS were detected on a triple quadrupole mass spectrometer (Xevo TQ-S Waters, Milford, MA, USA). CS, DS and HS were ionized by electrospray ionization (ESI) in positive mode, whereas negative mode was used for KS. Data were acquired with MassLynx (version 4.1, Waters) and processed with TargetLynx, software included in the MassLynx package.

**Table S2:** SRM parameters for all GAGs and their dedicated IS

| **Name** | **Precursor (*m/z*)** | **Fragment (*m/z*)** | **Cone (V)** | **Dwell (s)** | **Collision energy (V)** | **RT (min)** |
| --- | --- | --- | --- | --- | --- | --- |
| Chondroitin | 426.0 | 236.0 | 36 | 0.03 | 12.0 | 2.20 |
| Chondroitin–^2^D_6_ IS | 432.0 | 239.0 | 36 | 0.03 | 12.0 | 2.20 |
| Dermatan | 426.0 | 236.0 | 36 | 0.03 | 12.0 | 1.60 |
| Dermatan–^2^D_6_ IS | 432.0 | 239.0 | 36 | 0.03 | 12.0 | 1.60 |
| Heparan | 406.0 | 245.0 | 22 | 0.08 | 29.0 | 3.16 |
| Heparan – ^2^D_6_ IS | 412.0 | 251.0 | 22 | 0.08 | 29.0 | 3.16 |
| LacNAc (S1) | 462.1 | 361.0 | 75 | 0.096 | 25.0 | 4.01 |
| LacNAc (S1) – ^13^C_6_ | 468.1 | 367.0 | 77 | 0.096 | 25.0 | 4.01 |
| LacNAc (S2) | 542.1 | 462.1 | 35 | 0.096 | 20.0 | 4.38 |
| LacNAc (S2) – ^13^C_6_ | 548.1 | 468.1 | 35 | 0.096 | 20.0 | 4.38 |

**Validation data**

Interday precision was for CS, DS, HS below 13% at low concentrations (2 g/molCr); below 10% at middle concentrations (20 g/molCr); and below 8% at high concentrations (200 g/molCr). For KS, interday precision was below 8% at low concentration (1 g/molCr) and below 5% at middle and high concentrations (4 g/molCr and 16 g/molCr). Recovery for all analytes was below +/- 10% at low concentrations; below +/- 14% at middle concentrations and below +/- 8% at high concentrations. Linearity was controlled using the 8-point calibration curve (separate injection).

**DMB photometric assay**

Total GAG concentrations were measured with modifications of published protocol.^3^ The assay was performed in untreated urine samples stored at -20 °C. Briefly, the DMMB stock reagent is prepared with 10.7 mg 1,9-Dimethylmethylene blue chloride (research grade, SERVA Electrophoresis GmbH Cat.No. 20335 ) dissolved in 55 mmol/L formiate buffer, the stock is stored in aliquots at -20 °C for up to one year. Shortly before the assay 30 mL of the DMMB stock reagent is mixed with 3 mL of 2 mol/L Tris (hydroxymethyl)-aminomethan solution to end up with a pH of 8.8. The calibration curve is done with chondroitin-6-sulfate sodium salt from shark cartilage (MERCK, Cat.No. 27043) at concentrations of 5 / 12.5 / 25 / 37.5 / 50 µg/mL in water. For the measurement 60 µL urine/water/standard, 90 µL water and 825 µL DMB reagent were mixed in cuvettes and after 5 min absorbance is measured at 520 nm. Based on the linear standard curve sample concentrations are calculated and samples above 37.5 µg/mL were diluted with water and again measured. All urine results were expressed in g/moL creatinine as the ratio of quantitative GAG to urine creatinine and evaluated age dependent according to Table S1. Performance of the assay was checked with internal controls in every assay and in external quality assurance schemes (ERNDIMQA-Special assays in urine, 8 samples / year).

**Table S3:** Mean, standard deviation and upper limits defined as “mean + 2SD” for total GAG by DMB photometric assay

| **Age group** | **Total GAG (g/mol Cr)** | | | |
| --- | --- | --- | --- | --- |
|  | **n** | **Mean** | **SD** | **Upper limit [Mean + 2SD]** |
| 0–1 y | 38 | 24.4 | 9.2 | 42.7 |
| 1–3 y | 34 | 15.5 | 5.1 | 25.7 |
| 3–7 y | 29 | 9.9 | 2.5 | 14.8 |
| 7–10 y | 19 | 7.8 | 1.4 | 10.5 |
| 10–14 y | 14 | 6.8 | 1.6 | 10.1 |
| 14–20 y | 13 | 4.1 | 1.6 | 7.3 |
| <20 y | 12 | 2.8 | 1.0 | 4.8 |

**Supplementary Results**

**
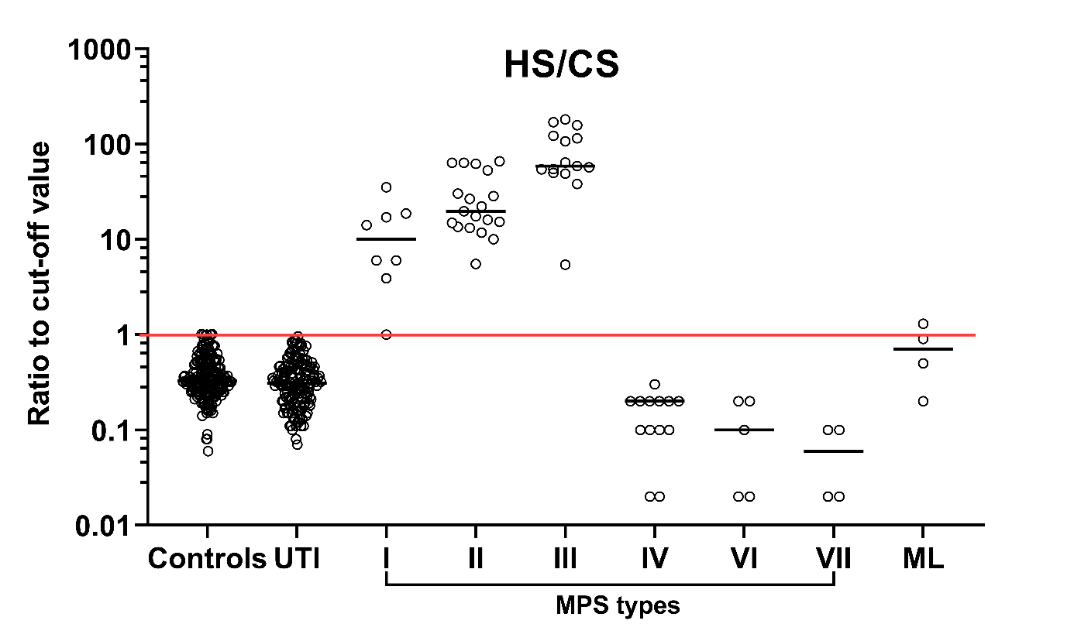
**

**Figure S1:** Ratio to cut-off values (>97.5 p) of HS/CS in urine samples of controls (n=183), urinary tract infection (UTI, n=153), and untreated patients (n=68) with mucopolysaccaridosis (MPS) type I, II, III, IV, VI, VII and mucolipidosis (ML).


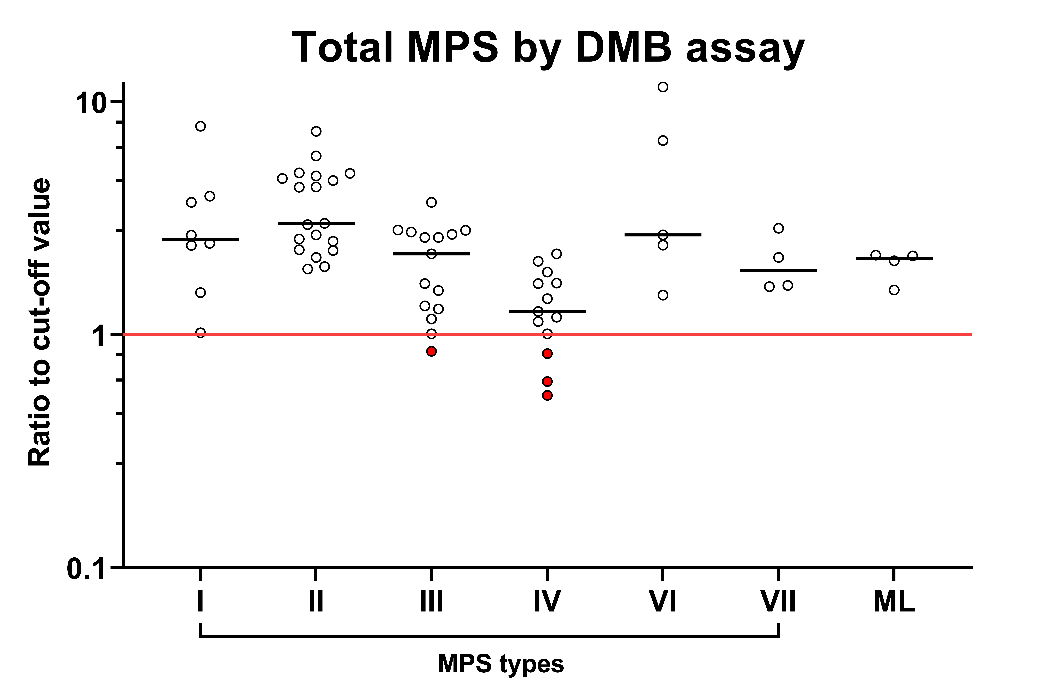


**Figure S2**: Ratio to cut-off value for total MPS by DMB photometric assay in treated and untreated MPS patient and ML urine samples.

**Table S4**: Median and 97.5 percentile of dermatan sulfate (DS), chondroitin sulfate (CS), heparan sulfate (HS), and keratan sulfate (HS) as well as cut-offs of specific ratios HS/DS and KS/HS for all age groups. The highest value measured in controls was taken as cut-off. HS/DS was not age-dependent so that the highest value amongst all age groups was used. Concentrations in g/mol Cr.

| **Age groups** | **DS** | | **CS** | | **HS** | | **KS** | | **HS/DS** | **KS/HS** |
| --- | --- | --- | --- | --- | --- | --- | --- | --- | --- | --- |
|  | **median** | **97.5 p.** | **median** | **97.5 p.** | **median** | **97.5 p.** | **median** | **97.5 p.** | **Cut-off** | **Cut-off** |
| 0-0.5 y (n=31) | 26.6 | 65.9 | 24.5 | 52.5 | 2.8 | 8.6 | 5.1 | 11.1 | 0.5 | 4.7 |
| 0.5-1 y (n=20) | 12.8 | 32.8 | 13.3 | 21.2 | 1.8 | 6.3 | 2.5 | 5.4 | 0.5 | 3.5 |
| 1-4 y (n=26) | 11.5 | 19.3 | 10.6 | 24 | 1.9 | 6.2 | 2.6 | 4.9 | 0.5 | 3.2 |
| 4-10 y (n=42) | 7.9 | 14.8 | 5.0 | 11.6 | 1.5 | 3.7 | 1.9 | 3.4 | 0.5 | 3.0 |
| 10-18 y (n=34) | 6.8 | 13.9 | 2.8 | 5.4 | 0.9 | 1.9 | 0.8 | 2.0 | 0.5 | 5.0 |
| >18 y (n=30) | 7.6 | 14.8 | 1.1 | 3.8 | 0.8 | 2.6 | 0.2 | 0.4 | 0.5 | 0.7 |

**Table S5:** Comparison of reference values

Zhang et al. ^1^, Concentrations in g/mol Cr.

| **Age groups** | **DS** | | | **CS** | | | **HS** | | | **KS** | | |
| --- | --- | --- | --- | --- | --- | --- | --- | --- | --- | --- | --- | --- |
|  | **mean** | **SD** | **97.5 p.** | **mean** | **SD** | **97.5 p.** | **mean** | **SD** | **97.5 p.** | **mean** | **SD** | **97.5 p.** |
| 0-0.5 y | 5.2 | 2.6 | 10.7 | 27.9 | 11.1 | 55.6 | 3.5 | 1.7 | 8.3 |  |  |  |
| 0.5-1 y | 3.1 | 1.3 | 6.6 | 14.8 | 5.1 | 26.6 | 2.4 | 1 | 4.7 |  |  |  |
| 1-4 y | 2.2 | 0.9 | 4.7 | 9.3 | 3.8 | 18.8 | 1.2 | 0.6 | 2.4 |  |  |  |
| 4-10 y | 2.2 | 0.9 | 4.3 | 5.2 | 1.9 | 10.2 | 0.7 | 0.3 | 1.7 |  |  |  |
| 10-18 y | 2.1 | 1 | 5.2 | 2.8 | 1.9 | 7.3 | 0.5 | 0.3 | 0.4 |  |  |  |
| >18 y | 2.1 | 1.1 | 4.6 | 1.3 | 0.5 | 2.5 | 0.5 | 0.2 | 1.2 |  |  |  |

Auray-Blais et al. ^4^, Concentrations in g/mol Cr.

| **Age groups** | **DS** | | | **CS** | | | **HS** | | | **KS** | | |
| --- | --- | --- | --- | --- | --- | --- | --- | --- | --- | --- | --- | --- |
|  | **mean** | **SD** | **97.5 p.** | **mean** | **SD** | **97.5 p.** | **mean** | **SD** | **97.5 p.** | **mean** | **SD** | **97.5 p.** |
| 0-2 y |  |  |  |  |  |  |  |  |  | 5.29 | 2.54 | 5.29 |
| 3-6 y |  |  |  |  |  |  |  |  |  | 2.86 | 0.85 | 2.86 |
| 7-10 y |  |  |  |  |  |  |  |  |  | 1.65 | 0.76 | 1.65 |
| 11-15 y |  |  |  |  |  |  |  |  |  | 1.19 | 0.73 | 1.19 |
| > 15 y |  |  |  |  |  |  |  |  |  | 0.30 | 0.11 | 0.30 |

Auray-Blais et al. ^2^, Concentrations in g/mol Cr.

| **Age groups** | **DS** | | | **CS** | | | **HS** | | |  | | |
| --- | --- | --- | --- | --- | --- | --- | --- | --- | --- | --- | --- | --- |
|  | **mean** | **SD** | **97.5 p.** | **mean** | **SD** | **97.5 p.** | **mean** | **SD** | **97.5 p.** |  |  |  |
| < 1 y | 25 | 4.8 |  | 35 | 8.5 |  | 5 | 1.2 |  |  |  |  |
| 1-3 y | 14 | 3.8 |  | 19 | 3.2 |  | 3 | 0.6 |  |  |  |  |
| 4-9 y | 11 | 1.9 |  | 12 | 2.4 |  | 2 | 0.4 |  |  |  |  |
| 10-17 y | 11 | 2.7 |  | 7 | 1.9 |  | 2 | 0.4 |  |  |  |  |
| > 18 y | 9 | 1.2 |  | 2 | 0.8 |  | 1 | 0.2 |  |  |  |  |

**References**

1. Zhang, H.*, et al.* Comparison of dermatan sulfate and heparan sulfate concentrations in serum, cerebrospinal fluid and urine in patients with mucopolysaccharidosis type I receiving intravenous and intrathecal enzyme replacement therapy. *Clin Chim Acta* **508**, 179-184 (2020).

2. Auray-Blais, C.*, et al.* UPLC-MS/MS detection of disaccharides derived from glycosaminoglycans as biomarkers of mucopolysaccharidoses. *Anal Chim Acta* **936**, 139-148 (2016).

3. de Jong, J.G., Wevers, R.A. & Liebrand-van Sambeek, R. Measuring urinary glycosaminoglycans in the presence of protein: an improved screening procedure for mucopolysaccharidoses based on dimethylmethylene blue. *Clin Chem* **38**, 803-807 (1992).

4. Auray-Blais, C., Lavoie, P., Maranda, B. & Boutin, M. Evaluation of urinary keratan sulfate disaccharides in MPS IVA patients using UPLC-MS/MS. *Bioanalysis* **8**, 179-191 (2016).
